# Sparse Hierarchical Representation Learning on Functional Brain Networks for Prediction of Autism Severity Levels

**DOI:** 10.3389/fnins.2022.935431

**Published:** 2022-07-07

**Authors:** Hyeokjin Kwon, Johanna Inhyang Kim, Seung-Yeon Son, Yong Hun Jang, Bung-Nyun Kim, Hyun Ju Lee, Jong-Min Lee

**Affiliations:** ^1^Department of Electronic Engineering, Hanyang University, Seoul, South Korea; ^2^Department of Psychiatry, Hanyang University Medical Center, Seoul, South Korea; ^3^Department of Artificial Intelligence, Hanyang University, Seoul, South Korea; ^4^Department of Pediatrics, Hanyang University College of Medicine, Seoul, South Korea; ^5^Division of Child and Adolescent Psychiatry, Department of Psychiatry, Seoul National University College of Medicine, Seoul, South Korea; ^6^Department of Biomedical Engineering, Hanyang University, Seoul, South Korea

**Keywords:** sparse hierarchical graph representation, ABIDE, ASD, functional brain network, graph neural network

## Abstract

Machine learning algorithms have been widely applied in diagnostic tools for autism spectrum disorder (ASD), revealing an altered brain connectivity. However, little is known about whether an magnetic resonance imaging (MRI)-based brain network is related to the severity of ASD symptoms in a large-scale cohort. We propose a graph convolution neural network-based framework that can generate sparse hierarchical graph representations for functional brain connectivity. Instead of assigning initial features for each node, we utilized a feature extractor to derive node features and the extracted representations can be fed to a hierarchical graph self-attention framework to effectively represent the entire graph. By incorporating connectivity embeddings in the feature extractor, we propose adjacency embedding networks to characterize the heterogeneous representations of the brain connectivity. Our proposed model variants outperform the benchmarking model with different configurations of adjacency embedding networks and types of functional connectivity matrices. Using this approach with the best configuration (SHEN atlas for node definition, Tikhonov correlation for connectivity estimation, and identity-adjacency embedding), we were able to predict individual ASD severity levels with a meaningful accuracy: the mean absolute error (MAE) and correlation between predicted and observed ASD severity scores resulted in 0.96, and *r* = 0.61 (*P* < 0.0001), respectively. To obtain a better understanding on how to generate better representations, we investigate the relationships between the extracted feature embeddings and the graph theory-based nodal measurements using canonical correlation analysis. Finally, we visualized the model to identify the most contributive functional connections for predicting ASD severity scores.

## Introduction

Autism spectrum disorder (ASD) is a complex neurodevelopmental disorder with increasing prevalence, with most recent statistics reporting that an estimated 1 out of every 59 children in the US has some form of ASD (Baio et al., 2018). Previous studies on the neurobiology underling the etiology and symptom presentation of ASD have been as heterogeneous and diverse as the behavioral phenotypes of ASD (Minshew and Williams, 2007). Recent neuroimaging studies have delineated the ASD brain as a representation of a typical organization of structural and functional brain networks (Uddin et al., 2013; Wadhera, 2021), affected not only by ASD core symptomatology but also by age, sex, ethnicity, and cognitive profile (Dosenbach et al., 2010; Tomasi and Volkow, 2012). ASD symptom severity has been reported to be associated with symptom trajectory, intensity of school services (e.g., number of services required), treatment response, and comorbidities (Zachor and Ben Itzchak, 2010; Adams et al., 2014; Andersen et al., 2017; Rosen et al., 2019). Therefore, determination of ASD severity may assist in planning individualized treatment plans, tracking treatment effects or disease progression, and providing insight into the neural substrates underlying ASD phenotypic heterogeneity (Moradi et al., 2017; Liu and Huang, 2020; Wadhera and Kakkar, 2021). Machine learning-based predictive modeling has recently been utilized to decode symptom severity from neuroimaging data (Sui et al., 2020; Wadhera et al., 2021); however, compared to binary classification, severity prediction may be more challenging as it requires the quantitative estimation of specific scores along a continuous behavioral measure, over a wide range, rather than just determining group membership (Shen et al., 2017; Sui et al., 2020). Although these models used neuroimaging measures like cortical thickness (Sato et al., 2013; Moradi et al., 2017), surface area (Pua et al., 2019), and functional connectivity (Uddin, 2014; Yahata et al., 2016; Lake et al., 2019; D’Souza et al., 2020; Liu and Huang, 2020; Pua et al., 2021) as features, putative findings have demonstrated a lack of consistency and reproducibility among them.

The recent success of convolutional neural networks (CNNs) in predicting problems associated with neurodevelopmental disorders has received significant attention (Khosla et al., 2019; Ronicko et al., 2020; Sherkatghanad et al., 2020). The convolution and pooling layers of the CNN models are mainly used to exploit the local meaningful features and spatial context, which are based on the spatial distribution of the Euclidean data [e.g., a 2-dimensional (2D)/3-dimensional (3D) grid image, and text]. CNN models are also applied to graph-structured data (e.g., functional/structural brain network), which can be considered as a generalized case of Euclidean data (LeCun et al., 2015). For example, Phang et al. (2019) applied a 2D CNN to brain connectivity data to investigate individuals with schizophrenia, and Al-Hiyali et al. (2021) classified ASD subtypes by using a CNN model with dynamic functional connectivity-based features. Ronicko et al. (2020) employed a one-dimensional CNN model as a diagnostic classifier using a flattened functional brain connectivity matrix.

Although brain connectivity data can be partially addressed by CNN, graph neural networks (GNNs), which consist of more generalized convolution and pooling operations, are considered more suitable for leveraging the topological locality of graphs (Kawahara et al., 2017; Lee et al., 2019; Wu et al., 2020). Several approaches have been suggested for generalizing convolution operations for graph data (Lee et al., 2019; Wu et al., 2020). Kipf and Welling (2016) proposed a simplified propagation rule using a graph convolutional network (GCN) layer via a localized first-order approximation of the Chebyshev filters on graphs (Kipf and Welling, 2016). Kawahara et al. (2017) modified conventional grid-shaped convolution filters into an edge-to-edge (E2E) filter that enabled spatial feature aggregation over a line graph with a K-hop of 1 (Kawahara et al., 2017). More recently, Ying et al. (2018) proposed an end-to-end graph differential pooling (Diff-Pool) method by training soft assignment vectors, thereby leveraging the hierarchical structure in graph data (Ying et al., 2018). Although the application of Diff-Pool to graph data has been well-established for some graph applications, a major limitation of Diff-Pool is the quadratic computational complexity of its soft assignment (Cangea et al., 2018). Alternatively, Lee et al. (2019) introduced a hierarchical self-attention graph pooling mechanism, which could compute nodal self-attention scores using GCN layers and adopt the top-rank selection method as a node-pooling strategy.

Two important aspects of GNNs, the initial node feature assignment and the graph pooling method, should be carefully considered when they are applied to the brain network domain. For feature assignment, previous studies of brain networks have suggested nodes as correspondences to the brain regions with inherently inconsistent initial features such as correlation profiles (Ktena et al., 2018), the coordinates of center voxels (Kim and Ye, 2020; Li et al., 2020) and one-hot encoded vectors (Kim and Ye, 2020). However, Kim and Ye (2020) showed that training a model with different node initialization strategies results in inconsistent latent representation, which affects its prediction performance (Van der Maaten and Hinton, 2008; Kim and Ye, 2020). Kawahara et al. (2017) proposed a CNN-based framework that automatically learns the appropriate assignment of node features to alleviate this issue of inconsistent representations (Kawahara et al., 2017). In their framework, the connectivity-based features were first embedded on the line graph by an E2E operation using the brain network matrix and then aggregated by the edge-to-node (E2N) layer for a subset of the line graph nodes (edges in the original graph) that were related to a specified node to obtain the corresponding nodal features. For the graph pooling method, conventional 2D convolution filter approaches such as the node-to-graph (N2G) layer, which globally pool all the nodal features in an inherently flat way, potentially ignore any sparse and hierarchical structures of graph-based data (Ying et al., 2018). As mentioned earlier, the progressive graph pooling method in GNNs is more effective with the hierarchical structures of brain networks. Moreover, some researchers argue that the node representation mechanism of E2N in the feature extractor is similar to that of the node embedding in Diff-Pool. The trainable soft assignment vector in Diff-Pool effectively learns node features and generates a coarsened adjacency embedding, thereby representing the relationship between each pair of nodes. Therefore, a better graph representation can be expected with a learning strategy that estimates the adjacency embedding among the nodes. To the best of our knowledge, no previous work has simultaneously applied automatic feature initialization and hierarchical pooling strategies to brain network data.

We hypothesized that the combination of an automatic nodal feature extractor and a sparse hierarchical self-attention graph pooling layer could learn an effective representation of brain network data related to ASD symptom severity, and that our graph-based deep learning model could identify the predictive edges that contribute the most to ASD symptom severity estimation based on large-sample data from multiple sites. We constructed a GNN that combined a feature extractor consisting of E2E and E2N layers to automatically assign node-level features and a hierarchical self-attention graph pooling network. Our proposed frameworks were tested on the Autism Brain Imaging Data Exchange (ABIDE), a multi-site resting-state functional magnetic resonance imaging (fMRI) database, to predict ASD symptom severity (ABIDE I and II)^1^ (Di Martino et al., 2014; Di Martino et al., 2017). We employed the Autism Diagnostic Observation Schedule (ADOS) to calibrate the severity score, Autism Diagnostic Interview–Revised (ADI-R) social, verbal, and restricted, repetitive, and stereotyped patterns of behavior (RRB) scores. We explored the effects of brain atlas by comparing various atlases (Shen et al., 2013; Schaefer et al., 2018; Khosla et al., 2019; Nozais et al., 2020), and we calculated the empirical covariance of the time series for each subject using the Pearson correlation coefficient and the ridge-regularized partial correlation (Tikhonov) (Pervaiz et al., 2020). To investigate whether the feature extractor represented the nodal features effectively, we performed a correlation analysis between the node features extracted by the feature extractor and the graph theory-based node measures. Because there are no existing gold standards that can be used to determine good performance in GNNs, we developed a canonical correlation analysis (CCA)-based multi-level analysis to investigate deep node feature representations (Hotelling, 1992). Finally, we visualized the results using the saliency map method to identify the predictive edges which are most useful for predicting ASD symptom severity (Simonyan et al., 2013).

## Materials and Methods

### Autism Brain Imaging Data Exchange Dataset and Participants

We utilized T1-weighted and resting-state fMRI data from an open-source ASD dataset named the Autism Brain Imaging Database Exchange (ABIDE I and II; see text footnote 1). The ABIDE databases, which consist of multisite protocols with a calibrated diagnostic status, are suitable for verifying the generality of the prediction model (Ronicko et al., 2020). ABIDE I yielded 539 individuals with ASD (age 7–64 years) recruited from across 17 international sites, and the ABIDE II has collected data from 521 individuals with ASD (age 5–64 years) across 19 sites (Di Martino et al., 2014; Di Martino et al., 2017). Of these samples, after visual verification of preprocessing quality and applying several criteria for clarifying the result (only right-handed individuals were included in this study), we were left with 196 and 249 quality MRI data with phenotypic information of ADOS severity and ADI-R scores, respectively (see the MRI Data Preprocessing section for the detailed procedure). The diagnostic methods, inclusion and exclusion criteria of participants, and sequence parameters for each site are available on the Supplementary Material, and the ABIDE website (Supplementary Tables 1, 2 and Supplementary Figure 1). All sites contributing to the ABIDE are required to confirm that their local ethics committee have approved the data collection, and data were fully anonymized by the Health Insurance Portable and Accountability Act (HIPAA) guidelines.

### Clinical Assessment of Autism Severity

As a score representing symptom severity, we used the calibrated severity score (CSS) of ADOS-G (“ADOS_GOTHAM_SEVERITY” column in ABIDE-I) and the Comparison Scores of ADOS-2 (“ADOS_2_SEVERITY_TOTAL” column in ABIDE-II). As the ADOS scores are highly correlated with age, cognitive abilities, and/or language skills, and the raw ADOS scores are not directly comparable across ADOS modules, Gotham et al. (2009) developed a standardized metric of ADOS, named CSS (Gotham et al., 2009), and the CSS has been incorporated into the updated ADOS-2 as comparison scores (Venker et al., 2014). A score of 1–2 indicates minimal to no evidence of ASD, whereas scores of 3–4 correspond to low, 5–7 to moderate, and 8–10 to high levels of ASD severity (Venker et al., 2014). We also utilized the subscale scores of the Autism Diagnostic Interview-Revised (ADI-R) (Rutter et al., 2003), namely the reciprocal social interaction total score, abnormalities in communication verbal score, and the restricted, repetitive, and stereotyped patterns of behavior score. Although the ADI-R scores have not been normalized according to age or sex, ADI-R and ADOS have been considered the “gold standard” in symptom evaluation of ASD (Lefort-Besnard et al., 2020), and a combination of ADOS and ADI-R assessments has been shown to improve diagnostic validity (Kim and Lord, 2012). Although a discrepancy in the ability of the ADOS to capture ASD symptoms cataloged in the DSM-5 has been suggested, the ADI-R is more relevant than the ADOS for encompassing the breadth of ASD symptoms as defined by DSM-5 (Mazefsky et al., 2013).

### MRI Data Preprocessing

To remove artifactual sources of resting-state fMRI data such as head motion, and hardware and physiology anomalies, preprocessing was performed using the Analysis of Functional NeuroImages (AFNI)^2^ toolkit (Cox, 1996). The first five volumes were discarded for each subject, and despiking was performed to ensure continuous data. Slice timing correction was performed, and rigid-body transformation was used to align all the scans to a base image, yielding six displacement parameters (translations and rotations for x-, y-, and z-axes). Additionally, the T1-weighted images were segmented into white matter (WM), gray matter (GM), cerebrospinal fluid, and background voxels using a neural network classifier framework and the derived tissue masks and the T1 volumes were co-registered to the fMRI using affine linear registration (Collins et al., 1995). In this step, 163 subjects with poor quality alignments and 104 subjects with inaccurate tissue masks were excluded. All the masks and volumes of the native space were spatially normalized to a standard MNI 152 template and resampled with an isotropic 2 mm size. The normalized fMRI volumes were smoothed using a 6 mm full width at half maximum (FWHM) Gaussian kernel. Anatomy-based regressors from the eroded WM, large ventricle mask, and motion parameters were used to remove the nuisance signal for each voxel (Jo et al., 2010). Specifically, the signals from the eroded WM regions were extracted from the local neighborhood of the voxel with a radius of 15 mm. Finally, the time scans that had the Euclidian norm of the first derivative of head motion (>0.25) was censored, and a bandpass filter (0.009 < f < 0.08) was applied to reduce noise. We excluded 481 samples which had a number of functional volumes with a motion norm >0.25 over the entire scan time.

### Functional Connectivity Matrix Construction

To define the functional brain network, we first extracted the average BOLD signals from each brain parcel after preprocessing the MRI data. Existing brain parcellations, including the AAL, SHEN, FIND, and MMP atlases, were used to derive the ROIs to evaluate the robustness of our prediction. Functional edge values can be calculated by constructing a covariance structure of time-series signals from the ROIs for each subject. First, we used Pearson’s correlation coefficients to estimate the pairwise covariance values among the ROIs, yielding a correlation between –1 and +1, as follows:


$$c_{i,j}^{P\,e\,a\,r\,s\,o\,n}={\left(\tilde{t^{i}}\right)}^{T}\,\tilde{t^{j}}/\sqrt{{\left(t^{i}\right)}^{T}\,t^{i}}\,\sqrt{{\left(t^{j}\right)}^{T}\,t^{j}}$$

$c_{i,j}^{P\,e\,a\,r\,s\,o\,n}$ is the connectivity value between node *i* and node *j*. *t*^*i*^ and $\tilde{t^{i}}$ is the time series and the demeaned time series of node *i*. The Tikhonov partial correlation, which implements inverse covariance estimation subject to a regularized l2 norm, was also used to define the functional connectivity as follows:


$$P^{T\,i\,k\,h\,o\,n\,o\,v}={\left(C+\rho \,I\right)}^{-1}$$

*P^Tikhonov^* and *C* are the estimated precision matrix and the empirical covariance of the time series. Here, the regularization parameter ρ was set to 0.1, in a heuristic manner. *I* is an identity matrix with a size of *n* × *n* (*n* is the number of nodes). The estimated covariance matrix was proportionally thresholded with a sparsity of 5% to remove noisy elements and normalized to zero mean and unit variance to reduce bias, resulting in an undirected and weighted matrix. Prior to training, the inter-site variability and the covariate effect of sex and age at MRI scan were removed using element-wise Combat harmonization^3^ and linear regression methods (Fortin et al., 2018).

### Sparse Hierarchical Representation of the Functional Brain Networks

**FIGURE 1:**
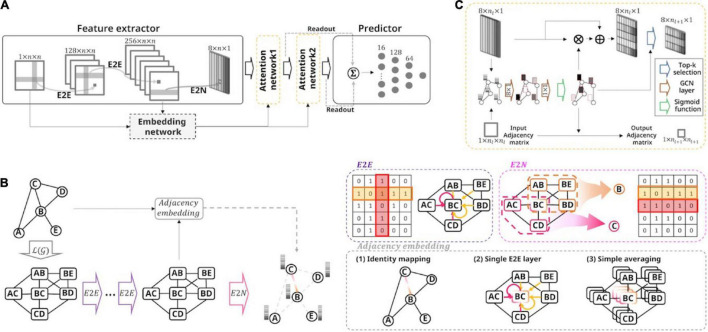
Overall framework of the proposed model and the toy example for the feature extractor. **(A)** Schematic of the model architecture. This model accepts the brain connectivity matrix as input and predicts the ASD severity score of all subjects. We employed the feature extractor followed by hierarchical self-attention graph pooling layers, embedding network, and fully connected network. The embedding network learned the adjacency embedding, which imposes information about the relationships among extracted node representations of the preceding feature extractor. **(B)** A toy example of the proposed feature extractor. With a sample graph (consisting of five nodes and five undirected-, and unweighted-edges), a stack of E2E layers embeds the connectivity features over a line graph of the original connectivity matrix. An E2N layer then aggregates the features to extract the node representations. Using input connectivity or embedded connectivity-based features, the embedding network define the adjacency matrix. There are three variants with the embedding layer: Identity, single E2E, and averaging method. **(C)** The self-attention graph pooling layer accepts the node-level features and embedded adjacency as input and learns a sparse graph representation. The GCN layers calculate the self-attention scores and then the scores were used to select the top *k* nodes. After selecting the nodes, the attention mechanism was performed using the survived nodes.

To specify the proposed ASD severity prediction model, we required four layers: a feature extractor, a graph attention network, an adjacency embedding network, and a prediction network (Figure 1A).

#### Fully Automatic Node Feature Extractor

First, the feature extractor consisted of two E2E layers and an E2N layer and represented nodal features using the input functional connectivity matrix (*g*). The hidden connectivity features were embedded by aggregating the adjacent node [over ℒ(*g*), representing the line graph of *g*] features using a cross-shaped E2E convolution kernel (Figure 1B). Formally, each E2E layer was a convolution operation, as follows:


$$f_{i,j}^{l+1,b}=lReLU\left(\sum_{a=1}^{d_{l}}\sum_{k=1}^{n}r_{k}^{l,a,b}f_{i,k}^{l,a}+c_{k}^{l,a,b}f_{k,j}^{l,a}\right),\forall b\in \left\{1,,d_{l+1}\right\}$$

where *f* is a *b*′*th* connectivity feature representation between node *i* and node *j* for layer *l*. *lReLU*(⋅) is the leaky rectified linear unit activation function with a leaky slope of 1/3. *d* is the number of feature maps in layer *l*, and n is the number of ROIs. [**r**^*l*,*a*,*b*^, **c**^*l*,*a*,*b*^] = **w**^*l*,*a*,*b*^ ∈ ℝ^2*n* × 1^ are the kernel parameters of the *b*′*th* convolution filter for layer *l*. Thus, the number of extracted feature maps does not change. The E2N layer further aggregated the embedded connectivity features of the subgraph related to the corresponding node to extract the node feature representation *e*^*l* + 1,*b*^ ∈ ℝ^*n* × 1^ (Figure 1B).


$$e_{i}^{l+1,b}=l\,R\,e\,L\,U\,\left(\sum_{a=1}^{d_{l}}\sum_{k=1}^{n}w_{k}^{l,a,b}\,f_{i,k}^{l,a}\right),\forall b\in \left\{1,\ldots ,d_{l+1}\right\}$$

where *e* is the *b*′*th* feature vector for node *i*, and similar to E2E, **w**^*l*,*a*,*b*^ ∈ ℝ^*n* × 1^ are the kernel parameters of the *b*′*th* convolution filter for layer *l*.

#### Hierarchical Graph Self-Attention Pooling

Second, the extracted node features were forwarded to a graph attention network by leveraging two stacks of graph self-attention layers. For each graph self-attention layer, we calculated the node self-attention scores by utilizing the GCN layers and selectively performing the pooling and attention methods for graphs, based on the scores using top-rank selection (Figure 1C). Specifically, given the input matrix*X*^*l*^ ∈ ℝ^*n* × *m*^ with *m*−dimensional node features, the attention layer for calculating the self-attention score *S*^*l*^ at layer *l* can be formulated as follows:


$$S^{l}=s\,i\,g\,m\,o\,i\,d\,\left(G\,C\,N\,\left(l\,R\,e\,L\,U\,\left(G\,C\,N\,\left(X^{l},A^{l};W^{l,0}\right)\right),A^{l};W^{l,1}\right)\right),\forall l\in \left\{1,,L\right\}$$

$$G\,C\,N\,\left(X,A;W\right)={\tilde{D}}^{-1/2}\,\tilde{A}\,{\tilde{D}}^{-1/2}\,X\,W$$

where the *W*^*l*,0^ ∈ ℝ^*m* × *p*^, and the *W*^*l*,1^ ∈ ℝ^*p* × 1^ are the trainable parameters in layer *l*. The $\tilde{A}=A+I,$ and the $\tilde{D}$ represent a self-connected adjacency matrix and a diagonal degree matrix, respectively. After deriving the self-attention score, the top-k selection algorithm was applied to coarsen the graph structure using the score *S*:


$${\dot{X}}^{l}=I\,n\,d\,e\,x\,\left(X^{l};S^{l},k\right),{\dot{A}}^{l}=I\,n\,d\,e\,x\,\left(A^{l};S^{l},k\right),{\dot{S}}^{l}\,=I\,n\,d\,e\,x\,\left(S^{l};S^{l},k\right)$$

$$X^{l+1}={\dot{X}}^{l}⊙{\dot{S}}^{l}+{\dot{X}}^{l},A^{l+1}={\dot{A}}^{l}$$

The indexing function *Index*(⋅; *S*, *k*) utilizes the top k score indices and selects the *k* nodes using the self-attention score *S*. The residual learning framework was used to leverage gradient flow using the proposed deep network (Woo et al., 2018). The residual output of the attention-pooling layer is calculated by applying the selected attention scores to the feature vector with element-wise multiplication ⊙, and is then added to the identity mapping of the input. To obtain optimized hyperparameters, such as the number of hidden feature maps *p*, the number of GCN layers *L*, and the selection ratio *k*, we implemented the replicated models with different values and evaluated the prediction performances (Supplementary Tables 3, 4). As a result, we set the hyperparameters as follows: *p* = 1, *L* = 2, and *k* = 1/2. Finally, the outputs of the stack of graph attention layers are summarized using the readout mechanism as follows:


$$\text{z}=\sum_{l=1}^{L}\left\lfloor 1/n^{l}\,\sum_{i=1}^{n^{l}}{\text{x}}_{i}^{l}∥\max_{1\leq i\leq n^{l}}{\text{x}}_{i}^{l}\right\rfloor$$

where ∥ denotes the concatenation operator and *L* is the number of last attention layers. *n*^*l*^ is the number of selected nodes, and ${\text{x}}_{i}^{l}$ is the feature vector of the *i*′*th* node in layer *l*.

#### Adjacency Embedding

The inputs to the sparse hierarchical self-attention graph pooling network were the two folds: (1) nodal feature vectors, and (2) an adjacency matrix representing the association among the nodes. To embed the adjacency matrix for the graph attention network, we suggested three possible strategies: identity, E2E, and averaging mapping methods. The adjacency embedding methods were formulated, respectively, as follows:


$$A_{i,j}=\left\{ \begin{array}{c} 1,i\,f\,s\,i\,g\,m\,o\,i\,d\,\left(R_{i,j}\right)\geq 0.5\\ 0,i\,f\,o\,t\,h\,e\,r\,w\,i\,s\,e \end{array}\right.$$

$$R_{i,j}^{i\,d\,e\,n\,t\,i\,t\,y}=f_{i,j}^{1},R_{i,j}^{E\,2\,E}=\sum_{a=1}^{d_{L}}\sum_{k=1}^{n}r_{k}^{L,a}\,f_{i,k}^{L,a}+c_{k}^{L,a}\,f_{k,j}^{L,a},R_{i,j}^{a\,v\,e\,r\,a\,g\,i\,n\,g}\,=1/d_{L}\,\sum_{a=1}^{d_{L}}f_{i,j}^{L,a}$$

The latent adjacency matrix *A* between node *i* and node *j* was represented by thresholding the connectivity-based feature map *R*, after applying sigmoid non-linearity. *d*_*L*_ is the number of feature maps in layer *L*, and [**r**^*L*,*a*^, **c**^*L*,*a*^] = **w**^*L*,*a*^ ∈ ℝ^2*n* × 1^ are the kernel parameters of the E2E convolution filter for layer *L*. *L* is the index of the last E2E layer (in this study, *L* = 2). Intuitively, we first used input functional connectivity to define the adjacency. The input functional connectivity matrix *f*^1^ can be used to embed the adjacency after applying the sigmoid non-linearity and thresholding with a value of 0.5 (identity-embedding method, Figure 1B). Furthermore, in line with the E2N framework of the preceding feature extractor, we developed two novel adjacency embedding variants that reflect the node feature representations with reasonable complexity (Figure 1B). The first adjacency embedding strategy is the E2E-embedding method, which applies an E2E kernel to the hidden connectivity features from the final E2E layer of the feature extractor to represent the relationships $R_{i,j}^{E\,2\,E}$among the nodes. The main concept of E2E-embedding is that high-level adjacency can be learned in a data-driven manner by aggregating feature representations of the subgraphs related to the corresponding node pair over the ℒ(*g*). Second, we introduced the averaging-embedding method to derive adjacency $R_{i,j}^{a\,v\,e\,r\,a\,g\,i\,n\,g}$ by averaging the hidden connectivity features in the final E2E layer of the feature extractor. This was done because the hidden connectivity features were already aggregated by applying the stacked E2E layers to the input connectivity matrix, and we expected that latent adjacency among nodes could be obtained by simply averaging it, even without any additional free parameters. Similar to the identity-embedding, the obtained adjacency of the two other methods (both E2E- and averaging-embedding) was used in the graph attention network by applying the sigmoid function and thresholding mechanisms. As a result, the graph attention network represented the graph features in a sparse hierarchical manner by using the embedded adjacency and the calculated node features.

#### Prediction Layer

Finally, the outputs of each self-attention graph layer were summarized in the readout layer, and the summation of the outputs of each readout layer was fed to the prediction network which consisted of fully connected layers (Figure 1A). The output vector of the hierarchical graph self-attention pooling **z** ∈ ℝ^2*d*_*L*_ × 1^ was used to predict the severity score in the fully connected layers consisting of two hidden layers with sizes of 128 and 64, respectively.

### Experimental Setting

We reported the mean absolute error (MAE), Pearson correlation coefficient (*r*), and corresponding statistical significance (*p*-value) between the predicted and observed ASD severity scores across various adjacency embedding networks. We used the BrainNetCNN, originally proposed by Kawahara et al. (2017) as a benchmarking model (26). The hyperparameters for the number of layers and dimension of the hidden representations for the BrainNetCNN are defined accordingly to match parameter which were used in our proposed models. We used a stochastic gradient descent (SGD) optimizer with an initial learning rate (α_0_) of 0.00001, which gradually decreased from the initial value to 0 each epoch using the following cosine function and a momentum of 0.9.


$$\alpha_{t}=1/2\,\left(1+\cos\left(t\,\pi /T\right)\right)\,\alpha_{0}$$

where α is the learning rate at *t*′*th* training epoch, and *T* is the total number of epochs (here, *T* = 1000). We evaluated the prediction performance using a 5-fold cross validation strategy. In each fold, 80% of the outer loop of the data was allocated to the training set, and the remaining 20% was used as the test set. In addition, we used an inner loop consisting of 90% training and 10% validation using the training set of the outer loop to optimize the hyperparameters of the model. The mini-batch size was eight, and we utilized the mean square error (MSE) as the loss function. All weights were initialized using the method described by He et al. (2015), and we used the standard weight decay algorithm with a regularization parameter of 0.0001 (He et al., 2015).

### Canonical Correlation Analysis

**FIGURE 2:**
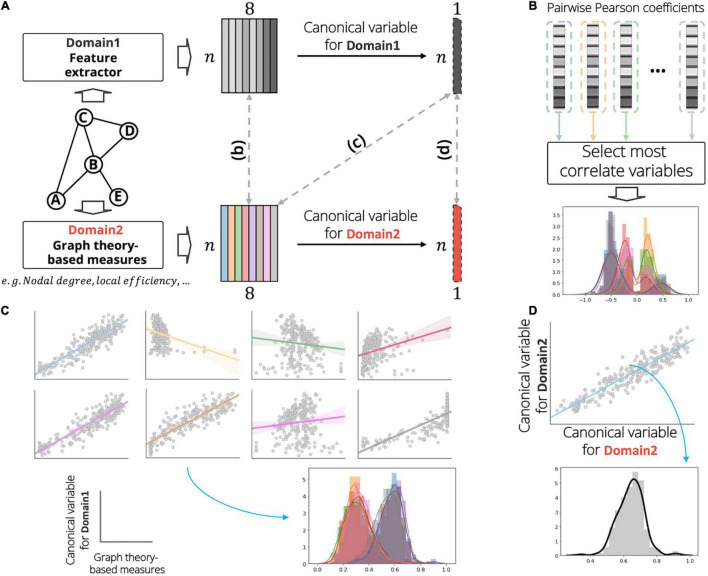
Graphical illustration of analysis on investigating the relationship between the extracted features (domain 1) and graph theory-based nodal measurements (domain 2). **(A)** Example of the analysis for identifying association between a sample graph data (consisting of five nodes and five undirected-, and unweighted-edges). In this figure, the number of nodal measurements and the number of extracted features were set to eight. At each domain, we calculate a linear combination to obtain the canonical variables. **(B)** First, we calculate pairwise univariate Pearson correlation coefficients between domains and select a most correlation variable for each nodal measurement. **(C)** Second, we calculate the Pearson correlation coefficients between the canonical variable of domain 1 and the measurements of domain 2. **(D)** Last, we derive the Pearson correlation coefficient between the canonical variables among domains. In the distribution plot, x-axis and y-axis indicate the correlation value and corresponding density, respectively. In the scatter plot of panel **(C)**, gray circles, solid lines, and shaded region demonstrate subject samples, linear regression line, and standard error for all samples, respectively.

For the correlation analysis, we calculated a total of eight node measures based on graph theory to investigate the relationships between the two domains (Figure 2A): nodal degree, clustering coefficient, local efficiency, betweenness centrality, eigenvector centrality, subgraph centrality, flow coefficient, and k core centrality. The CCA is useful in compactly investigating the linear relationship between two sets of multivariate features *X*_1_ ∈ ℝ^*n* × *p*^, and *X*_2_ ∈ ℝ^*n* × *q*^, by estimating the weight vectors *w*_1_ ∈ ℝ^*p* × 1^, and *w*_2_ ∈ ℝ^*q* × 1^ which maximize the correlation between the orthogonal linear combinations of the variables (Grellmann et al., 2015):


$$\max_{w_{1},w_{2}}c\,o\,r\,r\,\left(X_{1}\,w_{1},X_{2}\,w_{2}\right)$$

where *corr*(⋅) denotes the Pearson correlation operator. We assessed the node features extracted from the proposed feature extractor using three different methods to establish trust in the proposed model. First, the pairwise univariate correlations between the graph theory-based node measures *X*_*gm*_ ∈ ℝ^*n* × *p*^ and the extracted node feature representation *X*_*nf*_ ∈ ℝ^*n* × *q*^ were calculated, and the representative association *r* ∈ ℝ^*p* × 1^ was selected for each graph measure (Figure 2B):


$$r_{i}=\max_{1\leq j\leq q}c\,o\,r\,r\,\left(x_{g\,m}^{i},x_{n\,f}^{j}\right),\forall i\in \left\{1,\ldots ,p\right\}$$

$x_{g\,m}^{i}=X_{g\,m}\,\left[:,i\right]$ is the graph measure vector of the *i*′*th* dimension, and $x_{n\,f}^{j}=X_{n\,f}\,\left[:,j\right]$ is the extracted feature vector of the *j*′*th* dimension. The result of the above analysis does not consider the high-level node feature representations of domain 1. Thus, we investigated the multivariate association between the node measures of domain 2 and the node features of domain 1 using CCA to distill the higher-level node feature information of domain 1. First, orthogonal linear combinations for the node features of domain 1 were calculated to maximize the correlation for each node measure of domain 2. Then, the distributions of Pearson correlation coefficients between the derived canonical variable and the corresponding node measures of domain 2 were obtained (Figure 2C). The canonical mode for node feature *X*_*nf*_ was calculated by estimating the canonical weight *w*_*nf*_ ∈ ℝ^*q* × 1^, and the relationships *r*^*c*1^ ∈ ℝ^*p* × 1^ were investigated for each graph measure *x*^*i*^_*gm*_:


$$r_{i}^{c\,1}=c\,o\,r\,r\,\left(x_{g\,m}^{i},X_{n\,f}\,{\hat{w}}_{n\,f}\right),w\,h\,e\,r\,e\,{\hat{w}}_{n\,f}\,=\max_{w_{n\,f}}c\,o\,r\,r\,\left(x_{g\,m}^{i},X_{n\,f}\,w_{n\,f}\right),\forall i\in \left\{1,\ldots ,p\right\}$$

Finally, we built an association between the orthogonal linear combination vectors of the graph measures *X*_*gm*_, and the extracted features *X*_*nf*_ (Figure 2D). Thus, the scalar *r*^*c*2^ is given by


$$r^{c\,2}=c\,o\,r\,r\,\left(X_{g\,m}\,{\hat{w}}_{g\,m},X_{n\,f}\,{\hat{w}}_{n\,f}\right),w\,h\,e\,r\,e\,\left[{\hat{w}}_{g\,m},{\hat{w}}_{n\,f}\right]\,=\max_{w_{g\,m},w_{n\,f}}c\,o\,r\,r\,\left(X_{g\,m}\,w_{g\,m},X_{n\,f}\,w_{n\,f}\right),\forall i\in \left\{1,\ldots ,p\right\}$$

## Results

### Evaluation of Performance for Autism Spectrum Disorder Symptom Severity Prediction

**FIGURE 3:**
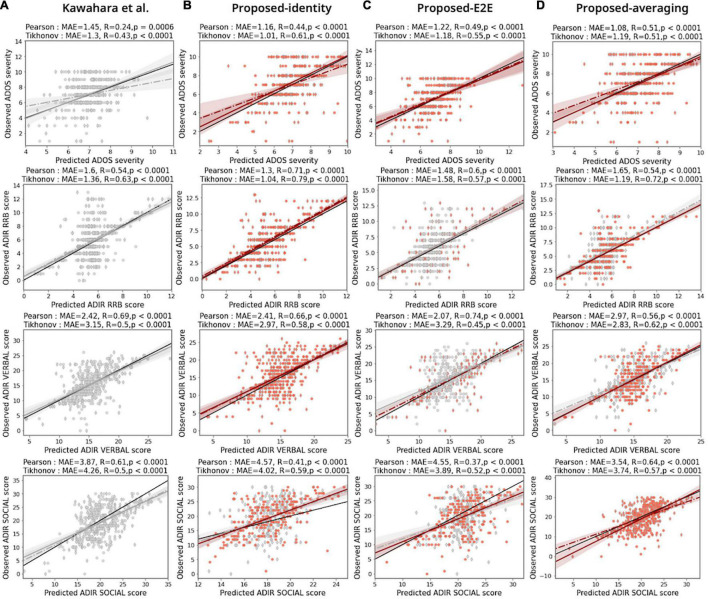
Prediction performances for the proposed model. Comparison of ASD prediction results for benchmarking BrainNetCNN model [Kawahara et al. (2017): panel **(A)**] and different configurations of proposed model [panels **(B–D)**] under two functional connectivity settings based on the Pearson correlation coefficient (diamond markers and dashed line of best fit) and the Tikhonov covariance (circle markers and solid line of best fit). MAE is the mean absolute error between predicted severity scores and observed severity scores. R and *p* indicate the Pearson correlation coefficient and corresponding statistical significance between predicted severity scores and observed severity scores, respectively. The colors of the markers and lines mean the increase (red) or decrease (gray) MAE of prediction compared with the benchmarking model. Black solid line in each panel indicates the line with slope of 1. The AAL atlas were used to define the node regions.

The sparse hierarchical graph representation framework for functional brain networks was built by stacking the feature extractor and hierarchical self-attention graph pooling networks. Figures 3A,B show the prediction results for different ASD severity scores of the benchmark model [BrainNetCNN; Kawahara et al. (2017), Figure 3A] and the proposed model with the baseline configuration (AAL atlas for node definition, Tikhonov correlation for connectivity estimation, and identity-adjacency embedding, Figure 3B), respectively. We observed that the proposed model achieved a MAE of 1.01, and 1.04, and an *r* = 0.61, and *r* = 0.79, *p* < 0.0001 for both cases, better than the benchmark model (MAE of 1.30, and 1.36, and *r* = 0.43, and *r* = 0.63, *p* < 0.0001 for both cases), for predicting the ADOS severity, and ADI-R RRB, respectively. Similarly, the proposed model performed better than the benchmark model (MAE of 3.15, and *r* = 0.50, *p* < 0.0001), which yielded a (MAE of 2.97, and *r* = 0.58, *p* < 0.0001) for predicting the ADI-R verbal. The same observation was made for predicting the ADI-R social score, which yielded a MAE of 4.02, with *r* = 0.59, *p* < 0.0001. In this case, the benchmark model achieved a MAE of 4.51, and an *r* = 0.50, *p* < 0.0001. In the above results, one consistent finding was that the proposed models always outperformed the benchmark model for ASD severity prediction tasks.

### Comparison of Prediction Performance Across Configurations of the Adjacency Embedding Network

Figures 3B–D show the prediction results of the proposed model with different adjacency embedding networks. We found that the proposed model with identity-embedding achieved the best performance for predicting both the ADOS severity score and the ADI-R RRB score (the MAE between the predicted and observed ADOS severity was 1.01, *r* = 0.61, *p* < 0.0001; MAE between predicted and observed ADI-R RRB score was 1.04, *r* = 0.79, *P* < 0.0001; Figure 3B, first two rows). Using the proposed model with E2E-embedding, we were able to accurately predict the ADI-R verbal scores with the same experimental setting (MAE = 2.07, r = 0.74, *P* < 0.0001; Figure 3C, third row). For predicting the ADI-R social score, the proposed model with the averaging-embedding framework achieved the best performance (MAE between predicted and observed ADI-R social was 2.54, *r* = 0.64, *p* < 0.0001; Figure 3D, fourth row).

### Comparison of Prediction Performance Across Methods to Define the Atlas and Edge for the Functional Brain Networks

We replicated these findings in the prediction analysis, wherein we defined the node parcels by using the AAL, FIND, SHEN, and MMP atlases (Figure 3 and Supplementary Figures 2–4). Our model achieved the highest prediction performance with the SHEN node atlas, Tikhonov connectivity, and identity adjacency embedding for predicting ADOS severity scores (MAE between predicted and observed ADOS severity was 0.96, *r* = 0.61, *p* < 0.0001). Similarly, we found that with respect to ADI-R RRB scores, the model achieved the best prediction performance with the AAL node atlas, Tikhonov connectivity, and identity adjacency embedding (MAE between predicted and observed ADI-R RRB was 1.04, *r* = 0.79, *p* < 0.0001). In the case of the ADI-R verbal and ADI-R social scores, our proposed models were associated with improvements in the prediction performances when utilizing the different adjacency embedding networks (the MAE between the predicted and observed ADI-R verbal was 1.84, *r* = 0.79, *p* < 0.0001, with HCP node atlas, Pearson connectivity, and E2E adjacency embedding; the MAE between the predicted and observed ADI-R social was 3.17, *r* = 0.69, *p* < 0.0001, with the SHEN node atlas, Tikhonov connectivity, and averaging adjacency embedding). In most cases, we found that the prediction performance of the proposed network variants outperformed the BrainNetCNN benchmark for all ASD severity scores across distinct adjacency embedding techniques, indicating that the functional brain network data were effectively represented using our sparse hierarchical model. Note that the proposed model variants required only a minor increase in the number of trainable parameters as compared to the BrainNetCNN benchmark model. This is because the network consumes a reasonable number of free parameters in the attention layers consisting of GCN layers (Lee et al., 2019).

### Toward Explainable Graph Representation of the Proposed Model: The Feature Extractor

**FIGURE 4:**
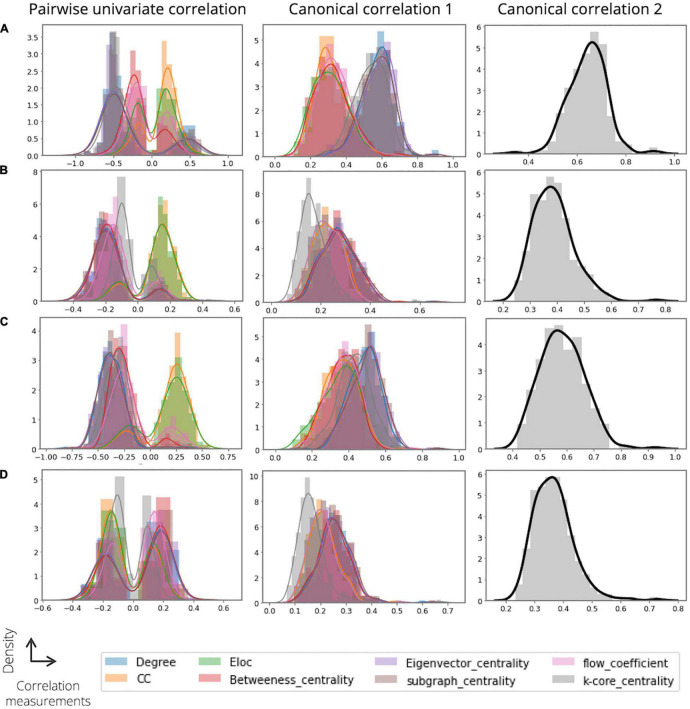
Results of analysis on investigating the relationship between the extracted features (domain 1) and graph theory-based nodal measurements (domain 2). For each analysis, we obtain and show the distribution of the correlations for **(A)** ASD severity score, **(B)** ADI RRB, **(C)** ADI verbal, and **(D)** ADI social. In the distribution plot, x-axis and y-axis indicate the correlation value and corresponding density, respectively.

Figure 4 shows examples of our analysis investigating the relationship between the extracted node features (domain 1) and the graph theory-based node measurements (domain 2). First, we obtained the pairwise univariate Pearson correlations among the domains for each individual and derived the distributions of the most highly correlated variables for each node measure (the maximal correlation coefficients). Regarding ADOS severity and ADI-R verbal scores, some node features of domain 1 showed comparatively high correlations with the nodal degree (mean ± std; 0.4913 0.1029 for ADOS severity and 0.3776 ± 0.1055 for ADI-R verbal; Figure 4A, left) and the eigenvector centrality (0.4963 ± 0.1080 for ADOS severity and 0.3794 ± 0.1115 for ADI-R verbal; Figure 4C, left). Furthermore, we observed that all the node measures of domain 2 had moderate correlations with the node features of domain 1 for the ADI-R RRB and social scores (Figures 4B,D, left). Second, we performed CCA to investigate the association between the linear combination of the extracted node embeddings and the node measures of domain 2. With regard to ADOS severity, we found that some node measures of domain 2, such as k-core centrality, nodal degree, eigenvector centrality, and subgraph centrality (0.5375 ± 0.0906, 0.5750 ± 0.0880, 0.5850 ± 0.0909, and 0.57120.0930, respectively) was highly correlated with the canonical variable of domain 1 (Figure 4A, middle). Similarly, for the models predicting the ADI-R RRB, verbal and social scores, nodal degree (0.2717 ± 0.0723 for RRB, 0.4846 ± 0.0876 for verbal, and 0.2539 ± 0.0618 for social), and some centrality measures of domain 2 revealed high linear associations with the canonical variable of domain 1 (Figures 4B–D, middle). Thus, Figures 4A–D (right) summarize the multivariate canonical correlations between the latent variables among domains for predicting ASD severity scores and thus provide a comprehensive overview of the extracted feature representations (0.6429 ± 0.0771 for ADOS severity, 0.3868 ± 0.0747 for ADI-R RRB, 0.5837 ± 0.0809 for ADI-R verbal, and 0.3664 ± 0.0694 for ADI-R social, respectively).

### Toward Explainable Graph Representation of the Proposed Model: Visualization

**FIGURE 5:**
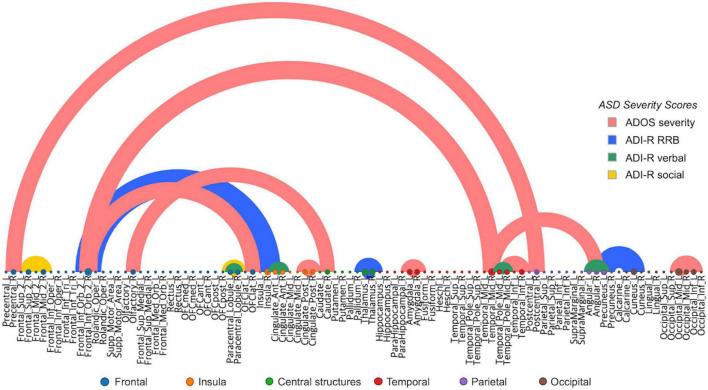
Most predictive connections for ASD severity score (Red), ADI RRB (Blue), ADI verbal (Green), and ADI social (Yellow), learned by our proposed model. For each severity score, the connections that have the top 10% magnitude were shown for clarity. Thickness of the line represents the magnitude of partial derivative.

We performed a saliency visualization method to map the most predictive functional brain connectivity values that were identified by our proposed model (Simonyan et al., 2013). A saliency map can be derived by calculating the gradient values of the prediction output with respect to the input connectivity. The partial derivatives were calculated for each subject and then averaged to obtain the group representative contribution matrix. For clarity, we empirically thresholded the edge contribution scores with a maximum intensity of 10% for each ASD severity score, as shown in Figure 5, Table 1, and Supplementary Video 1. The semicircular edges show the connections for each pair of regions (identified most contributing regions were listed in Table 1) which have a large contribution score. The labels in horizontal axis show the list of node regions. We found that the fronto-temporal and fronto-caudate connections, as well as interhemispheric connections within the limbic system including the left and right posterior cingulate cortex and amygdala, were selected to predict the ADOS severity score. Intrahemispheric connections in the temporal lobe and also temporo-angular connections, as well as connections between the precentral and postcentral gyrus, interhemispheric and intrahemispheric connections within the occipital gyrus were found to predict ADOS scores. Similarly, functional edges from the precuneus to cuneus, fronto-insular connections, interhemispheric connections of the thalamus of limbic system were selected for prediction of ADI-R RRB scores, edges from the anterior cingulate gyrus, temporal pole and paracentral lobule were selected for predicting ADI-R verbal scores. These results are consistent with those of previous studies suggesting that areas such as the fronto-limbic and the social brain play the most important role in analyzing ASD patients (Koshino et al., 2008; Perez et al., 2016)

**TABLE 1:** Most predictive connections for the severity of ASD based on ADOS and ADI.

	Region 1	Region 2	PD value
	
	ADOS		
1	Frontal_Inf_Orb_2_R	Temporal_Mid_R	0.027
2	Temporal_Pole_Mid_R	Temporal_Inf_R	0.026
3	Occipital_Mid_L	Occipital_Inf_L	0.026
4	Amygdala_L	Amygdala_R	0.026
5	Cingulate_Post_L	Cingulate_Post_R	0.025
6	Frontal_Inf_Orb_2_R	OFClat_R	0.023
7	Precentral_R	Postcentral_R	0.023
8	Angular_R	Temporal_Mid_R	0.022
9	Occipital_Mid_L	Occipital_Mid_R	0.022
10	Olfactory_R	Caudate_R	0.022

	**ADI-R RRB**		

1	Frontal_Inf_Orb_2_R	Insula_R	0.037
2	Cuneus_L	Precuneus_L	0.031
3	Thalamus_L	Thalamus_R	0.031

	**ADI-R verbal**		

1	Cingulate_Ant_L	Cingulate_Ant_R	0.076
2	Temporal_Pole_Mid_L	Temporal_Pole_Mid_R	0.073
3	Paracentral_Lobule_L	Paracentral_Lobule_R	0.063
4	Angular_L	Precuneus_L	0.062

	**ADI-R social**		

1	Frontal_Sup_2_R	Frontal_Mid_2_R	0.103
2	Paracentral_Lobule_L	Paracentral_Lobule_R	0.102

*PD value denote the partial derivatives for the saliency map analysis. ASD, autism spectrum disorder; ADOS, autism diagnostic observation schedule; ADI-R, autism diagnostic interview-revised; RRB, restricted, repetitive, and stereotyped patterns of behavior.*

## Discussion

### Potential Reasons Why the Proposed Model Accurately Predicts Autism Spectrum Disorder Severity

We predicted ASD symptom severity using the ABIDE database, utilizing the fully automatic nodal feature extractor and the sparse hierarchical graph representation framework to encode the brain’s functional connectivity. In the proposed frameworks, there are two key factors which may have led to the proposed models’ reliable prediction performances. First, the automatic feature extractor seems to play a significant role in the prediction tasks. Encouraged by the success of machine learning in the detection and classification of neurodevelopmental disorders, a number of methods that leverage machine learning-based models (Sato et al., 2013; Moradi et al., 2017; Liu and Huang, 2020), artificial neural networks (D’Souza et al., 2020), and CNNs (Khosla et al., 2019) to encode the neuroimaging data of ASDs have been developed in parallel. Unfortunately, the sparse and hierarchical structure of graph data which impose a topological locality cannot be considered by applying these methods (Kawahara et al., 2017). Therefore, although these models achieved a modest range of performance, yielding a MAE of 1.36∼2.53, and *r* of 0.36∼0.51 for predicting the ADOS severity, there remains a need for GNN-based approaches to improve the overall prediction performance. To solve this problem, Jiang et al. (2020) proposed a GNN approach involving two GCNs for modeling the population graph, which employs an individual node feature as vectorized functional connectivity data (Jiang et al., 2020). However, the flat representations of the vectorized functional connectivity for the node still ignored the graph-structured data and can result in degradations in the performance of the model (Ying et al., 2018). Even though research on individual brain networks considering a split of a parcel on brain regions as node representation were introduced, they inconsistently initialized the input node features, potentially ignoring the major benefit of the deep learning models. We fully addressed these issues by combining the proposed feature extractor suggested by Kawahara et al. (2017). Without any unnecessary feature initialization procedure, our proposed model achieved favorable prediction performance (MAE of 1.01, and *r* of 0.61, *p* < 0.0001 for predicting the ADOS severity) with reasonable computational complexity using a combination of the E2E and E2N networks.

The second key factor contributing to our reliable prediction performances was the sparse hierarchical self-attention graph-pooling networks. Recent studies suggest that brain networks can be substantial biomarkers for ASD and that these networks exhibit a small-world topology with hierarchical organizations dominated by a set of network hubs (He and Evans, 2010; Ronicko et al., 2020). Although the stacking of E2E layers introduced by Kawahara et al. (2017) enables the learning of these topology-based patterns, the standard convolutional layer in an N2G layer over the extracted graph representation does not consider the hierarchical structure of the brain network in the BrainNetCNN (Kawahara et al., 2017; Ying et al., 2018). Thus, we considered these inherent structures of the brain network as we adapted the sparse hierarchical graph representation paradigm by combining self-attention layers with the hierarchical readout method (Cangea et al., 2018; Lee et al., 2019).

### Effect of the Adjacency Embedding Network

We described the need to embed the latent adjacency among nodes and the frameworks for deriving it, as inspired by Diff-Pool (Ying et al., 2018). The trainable soft-assignment vector of Diff-Pool embeds the coarsened node features and adjacency information simultaneously to create a hierarchical representation of the graph-structured data. More specifically, they aggregated the node features from the previous layer to extract the coarsened node representations. Similarly, the E2N of our feature extractor aggregated the node embeddings of line graph ℒ(*g*), which was represented by the preceding E2E layers to extract the node features. Therefore, we expected to see a greater advantage of the graph representation when applying the adjacency embedding network, which learns a meaningful structure by embedding a latent adjacency, to our model. The derivation of latent adjacency using the input connectivity or embedded connectivity features along the line graph ℒ(*g*) were thus formulated. Furthermore, increased literature on graph-structured data such as point clouds have demonstrated that dynamic graph convolution on latent adjacency structure based on the node features can represent graphs more effectively (Simonovsky and Komodakis, 2017; Zhao et al., 2022). For example, Zhao et al. (2022) proposed the functional connectivity-based diagnostic GNN model to classify the ADHD status by utilizing the dynamic convolution layer using the top-k Euclidean distance between nodal hidden representations of functional brain networks (Zhao et al., 2022). Similarly, the latent adjacency embedding networks contribute to enhancing the generalizability and further improving the prediction performance of our model. Therefore, our sparse hierarchical graph representation model outperformed the BrainNetCNN benchmark model for ASD severity prediction and provided rich graph representations of the brain functional network data.

### Effect of Defining the Atlas and Edge for the Functional Brain Networks

The motivation behind using various brain atlases and edges for constructing functional connectivity is grounded in previous studies suggesting that the choice of these factors can impact the prediction performance of models. Shen et al. (2013) highlighted the importance of defining meaningful, functionally homogeneous regions as nodes, and Fornito et al. (2010) showed that there is significant variability across the scales of the regions of interest (ROIs) (Fornito et al., 2010; Shen et al., 2013). Thus, we applied four different atlases with various scales and algorithms to identify ROIs. The AAL atlas is composed of 90 Brodmann-based regions, and the FIND atlas is composed of 90 functional subunits based on group independent component analysis (ICA). The SHEN atlas produces 278 functionally coherent and reproducible regions using the groupwise clustering algorithm, and lastly the MMP atlas is composed of 374 regions based on multimodal imaging. Furthermore, it may be interesting to consider methods for estimating functional connectivity (edge). Although the Pearson correlation coefficient is typically used to derive the network edges, this technique does not adequately distinguish between direct and indirect connections among nodes (Pervaiz et al., 2020). We additionally defined the partial correlation by calculating the regularized Tikhonov connectivity to derive the functional connectivity matrix (Pervaiz et al., 2020). Therefore, the proposed models with various configurations perform at least comparable to, but mostly better than, the benchmark model for predicting ASD severity. In particular, a simple averaging-adjacency embedding network is more suitable when dealing with a small number of ROIs (e.g., AAL and FIND) for predicting the ADI-R RRB scores. In contrast, embedding relationships among a large number of ROIs using the E2E-adjacency embedding network seems to be more effective for SHEN or MMP atlases. In the case of network edges, the models using the Tikhonov connectivity almost always yielded a favorable prediction performance.

### Interpretation of the Node Representation

We also conducted an additional CCA-based analysis focusing on the represented node features extracted by the feature extractor to further elucidate the results derived from our proposed model. Even though our univariate pairwise correlation results showed the associations between extracted node features and the graph theory–based node measures in an intuitive manner, this method may be somewhat disadvantageous because it cannot account for high-level feature representations. Thus, we adopted CCA variants to leverage the high-level characteristics of the extracted node features and showed the linear relationships between the features and the node measures. Our results showed the inter-domain relationship between the extracted node embeddings and the node measures and thus provide insight into the feature extractor of our model. We believe that these results provide evidence that our model is an effective strategy to explain graph neural networks and to reason about their strong prediction performances.

### Visualization of the Model

An important goal of machine-learning tools in neuroimaging is to generate novel insights linking imaging biomarkers with disease or phenotypic traits. A detailed summary of previous studies that focused on prediction of ASD severity using machine learning or deep learning methods is presented in Supplementary Table 5. ADOS severity score represents the overall severity of ASD and was related to various regions in the brain, whereas ADI-R subscale scores correlates to specific symptom domains and the related regions were more focused. Various temporal-related and frontal-related resting state functional connections were related to ADOS severity, ADI-R verbal and social scores, as was consistent to findings of other studies (Liu and Huang, 2020). The temporal and frontal lobe are associated with advance cognitive, social and communication functions (Chomiak and Hu, 2013), whose functional abnormalities can cause the core symptoms of ASD (Verly et al., 2014; Yang et al., 2017). Temporal lobe dysfunction is primarily involved in speech formation and understanding, supporting social interactions, and higher order cognitive processing. High predictive values of connections related to the salience network (SN), default mode network (DMN), and sensory motor network (SMN) were reported in this study (see the Table 1), as was the case in several previous studies (Uddin, 2014; Pua et al., 2019; Liu and Huang, 2020; Pua et al., 2021). The DMN, which is consisted of the posterior cingulate cortex (PCC), precuneus and angular gyrus, was related to ADOS, ADI-R RRB and ADI-R verbal scores. The DMN plays a vital role in socially relevant stimuli because of its involvement in the mentation of self-reflective thought and in the consideration of the perspective of others (Padmanabhan et al., 2017). Some studies have reported that widely decreased resting state functional connections in the DMN in ASD contributes to the core deficit of ASD and also has a great influence on symptoms severity (Assaf et al., 2010; Weng et al., 2010; Jung et al., 2014). The SN is consisted of the anterior cingulate cortex (ACC) and insula, and was related to ADI-R RRB and verbal scores in this study. Reduced salience to social cues is coupled with poorer social functioning and increased visual fixation on inanimate objects (Klin et al., 2002). Atypical increased salience has been implicated in ASD symptomatology like hypersensitivity to sensory stimuli, or stereotypic and restricted behavior and interests (Wiggins et al., 2009). Moreover, regions belonging to the SN are involved in the maintenance of task sets during goal-directed behavior (Dosenbach et al., 2007), and an excess can contribute to restricted and repetitive behaviors. The SMN is a large-scale network that primarily includes the postcentral and precentral regions and extends to the supplementary motor area, and SMN-related connections were found to be related to ADOS severity and ADI-R social scores in this study. This is in line with recent studies showing that sensorimotor skills are associated with ASD symptom severity (Tavassoli et al., 2014; Hannant et al., 2016). Mosconi and Sweeney (2015) mentioned that sensorimotor deficits occur before social and communication deficits and Hannant et al. (2016) mentioned that sensorimotor deficits can lead to impairment of advanced functions, such as social, communicative and emotional development.

## Limitations and Conclusion

The results were replicated across the prediction experiments based on the different strategies of network construction (e.g., defining the node parcel and edge values), and we administered multiple variants of the model with respect to adjacency embedding methods. Further studies are warranted to identify the optimized guidelines for these variants of the proposed model to apply them to various prediction tasks. We can also expand our results by applying these models to neurological disorders characterized by aberrant connectivity, such as schizophrenia and ADHD. This study had a few limitations. First, ADI-R scores were not normalized according to age or sex. Moreover, recent studies have suggested differential brain connectivity patterns in individuals with high-functioning and low-functioning ASD. Although the majority of participants in the ABIDE database have an IQ above 70, the IQ level may be a source of bias for our results. As we did not consider the effect of IQ in our model, further studies are warranted to decipher the impact of IQ on ASD severity prediction. Another avenue for refinement is to leverage anatomical network information, such as the structural and morphological brain networks. Structural imaging modalities, including diffusion-weighted imaging and T1 weighted imaging-based gray matter density maps, are typically used to derive the above-mentioned brain networks. Integration of anatomical and functional connectivity will contribute to shaping a more comprehensive picture of complex and highly heterogeneous neurodevelopmental disorders, such as ASD (D’Souza et al., 2020).

In this paper, we propose a sparse hierarchical graph-representation framework for brain functional connectivity. To our knowledge, this is the first study to use a graph-based deep learning model to predict ASD severity. Our proposed model surpassed benchmarking published models for the prediction tasks of ASD severity scores without any explicit feature assignment. We extended the training of graph representation by applying a sparse hierarchical self-attention pooling network, and then investigated the embedded features and predictive components of the connectivity data. Our approach highlights the potential of sparsity and hierarchy in the mutually interconnected brain regions, which may facilitate individualized prediction of disease progression with increasing precision in various neurologic disorders.

## Data Availability Statement

Publicly available datasets were analyzed in this study. This data can be found here: http://fcon_1000.projects.nitrc.org/indi/abide/.

## Author Contributions

J-ML: conceptualization, methodology, and supervision. HL: conceptualization, supervision, and writing — original draft. HK: methodology, software, formal analysis, writing — original draft, and visualization. JK: methodology, visualization, data curation, and writing — original draft. S-YS: software and methodology. YJ: methodology and formal analysis. B-NK: methodology and data curation. All authors contributed to the article and approved the submitted version.

## Conflict of Interest

The authors declare that the research was conducted in the absence of any commercial or financial relationships that could be construed as a potential conflict of interest.

## Publisher’s Note

All claims expressed in this article are solely those of the authors and do not necessarily represent those of their affiliated organizations, or those of the publisher, the editors and the reviewers. Any product that may be evaluated in this article, or claim that may be made by its manufacturer, is not guaranteed or endorsed by the publisher.

## Footnotes

1
http://fcon_1000.projects.nitrc.org/indi/abide/

2
http://afni.nimh.nih.gov/afni

3
https://pypi.org/project/pycombat/
