# Supplementary material for: Sparse Hierarchical Representation Learning on Functional Brain Networks for Prediction of Autism Severity Levels
Source: Front Neurosci. 2022 Jul 7;16:935431. doi: 10.3389/fnins.2022.935431 (PMC9301472; doi:10.3389/fnins.2022.935431)
Supplement: Supplementary file 1 [file Data_Sheet_1.docx]

**Supplementary Information**

**Tables**

**Table S1. Participant information for the study with ADOS severity.**

|  | **Site** | **N** | **Sex: M/F** |
| --- | --- | --- | --- |
|  | **ABIDE1** |  |  |
| 1 | YALE | 16 | 10/6 |
| 2 | UM_2 | 5 | 4/1 |
| 3 | UCLA_1 | 3 | 3/0 |
| 4 | STANFORD | 7 | 6/1 |
| 5 | SDSU | 3 | 3/0 |
| 6 | KKI | 11 | 10/1 |
|  | **Total for ABIDE1** | 45 | 36/9 |
|  | **ABIDE2** |  |  |
| 1 | UCLA_1 | 9 | 9/0 |
| 2 | UCD_1 | 14 | 12/2 |
| 3 | U_MIA_1 | 7 | 6/1 |
| 4 | SU_2 | 19 | 18/1 |
| 5 | SDSU_1 | 22 | 17/5 |
| 6 | OHSU_1 | 30 | 28/1 |
| 7 | NYU_1 | 18 | 16/2 |
| 8 | NYU_2 | 9 | 9/0 |
| 9 | KKI_1 | 13 | 11/2 |
| 10 | IP_1 | 6 | 3/3 |
| 11 | GU_1 | 4 | 4/0 |
|  | **Total for ABIDE2** | 151 | 134/17 |
|  | **Total** | 196 | 170/26 |

**Table S2. Participant information for the study with ADI-R severity.**

|  | **Site** | **N** | **Sex: M/F** |
| --- | --- | --- | --- |
|  | **ABIDE1** |  |  |
| 1 | YALE | 13 | 9/4 |
| 2 | UM_2 | 6 | 5/1 |
| 3 | UCLA_1 | 3 | 3/0 |
| 4 | TRINITY | 24 | 24/0 |
| 5 | STANFORD | 12 | 10/2 |
| 6 | SDSU | 9 | 8/1 |
| 7 | PITT | 11 | 8/3 |
| 8 | OHSU | 10 | 10/0 |
| 9 | KKI | 11 | 10/1 |
| 10 | CALTECH | 9 | 6/3 |
|  | **Total for ABIDE1** | 108 | 93/15 |
|  | **ABIDE2** |  |  |
| 1 | TCD_1 | 11 | 11/0 |
| 2 | SDSU_1 | 22 | 17/5 |
| 3 | OILH_2 | 4 | 4/0 |
| 4 | OHSU_1 | 31 | 26/5 |
| 5 | NYU_1 | 17 | 15/2 |
| 6 | NYU_2 | 9 | 9/0 |
| 7 | KKI_1 | 25 | 19/6 |
| 8 | IP_1 | 7 | 5/2 |
| 9 | GU_1 | 10 | 10/0 |
| 10 | ETH_1 | 5 | 5/0 |
|  | **Total for ABIDE2** | 141 | 121/20 |
|  | **Total** | 249 | 214/35 |

**Table S3. Summary of the MAE between the predicted- and observed ADOS severity scores for hyperparameter optimization.** $\boldsymbol{p}$, and $\boldsymbol{L}$ correspond to the number of hidden state features, and the number of GCN layers in a self-attention layer, respectively. $\boldsymbol{K}$ indicates the selection ratio in the top-$\boldsymbol{k}$ pooling layer. In this case, the AAL atlas and the Pearson correlation coefficient were used to define the connectivity.

|  | $\boldsymbol{K=1/2}$ | | | $\boldsymbol{K=1/4}$ | | |
| --- | --- | --- | --- | --- | --- | --- |
|  | $\boldsymbol{L=1}$ | $\boldsymbol{L=2}$ | $\boldsymbol{L=3}$ | $\boldsymbol{L=1}$ | $\boldsymbol{L=2}$ | $\boldsymbol{L=3}$ |
| $\boldsymbol{p=1}$ | 1.356 | 1.156 | 1.392 | 1.379 | 1.273 | 1.392 |
| $\boldsymbol{p=2}$ | 1.327 | 1.222 | 1.226 | 1.378 | 1.267 | 1.296 |
| $\boldsymbol{p=4}$ | 1.462 | 1.300 | 1.402 | 1.185 | 1.340 | 1.448 |
| $\boldsymbol{p=8}$ | 1.414 | 1.320 | 1.486 | 1.353 | 1.359 | 1.326 |
| $\boldsymbol{p=None}$ | 1.301 | 1.262 | 1.406 | 1.320 | 1.417 | 1.378 |

MAE, mean absolute error; ADOS, autism diagnostic observation schedule; GCN, graph convolutional network; AAL, automated anatomical labelling.

**Table S4. Summary of the Pearson correlation coefficient (*R*) and corresponding statistical significance (*p*) between the predicted- and observed ADOS severity score for hyperparameter optimization.** $\boldsymbol{p}$, and $\boldsymbol{L}$ correspond to the number of hidden state features, and the number of GCN layers in a self-attention layer, respectively. $\boldsymbol{K}$ indicates the selection ratio in the top-$\boldsymbol{k}$ pooling layer. In this case, the AAL atlas and the Pearson correlation coefficient were used to define the connectivity.

|  | $\boldsymbol{K=1/2}$ | | | $\boldsymbol{K=1/4}$ | | |
| --- | --- | --- | --- | --- | --- | --- |
|  | $\boldsymbol{L=1}$ | $\boldsymbol{L=2}$ | $\boldsymbol{L=3}$ | $\boldsymbol{L=1}$ | $\boldsymbol{L=2}$ | $\boldsymbol{L=3}$ |
| $\boldsymbol{p=1}$ | *R* = 0.6214  *p* < 0.0001 | *R* = 0.6459  *p* < 0.0001 | *R* = 0.5964  *p* < 0.0001 | *R* = 0.5661  *p* < 0.0001 | *R* = 0.6650  *p* < 0.0001 | *R* = 0.5516  *p* < 0.0001 |
| $\boldsymbol{p=2}$ | *R* = 0.6023  *p* < 0.0001 | *R* = 0.6318  *p* < 0.0001 | *R* = 0.6340  *p* < 0.0001 | *R* = 0.5750  *p* < 0.0001 | *R* = 0.5823  *p* < 0.0001 | *R* = 0.5715  *p* < 0.0001 |
| $\boldsymbol{p=4}$ | *R* = 0.5179  *p* < 0.0001 | *R* = 0.5803  *p* < 0.0001 | *R* = 0.6079  *p* < 0.0001 | *R* = 0.6466  *p* < 0.0001 | *R* = 0.5703  *p* < 0.0001 | *R* = 0.5499  *p* < 0.0001 |
| $\boldsymbol{p=8}$ | *R* = 0.5835  *p* < 0.0001 | *R* = 0.5813  *p* < 0.0001 | *R* = 0.5156  *p* < 0.0001 | *R* = 0.5810  *p* < 0.0001 | *R* = 0.5788  *p* < 0.0001 | *R* = 0.5864  *p* < 0.0001 |
| $\boldsymbol{p=None}$ | *R* = 0.5992  *p* < 0.0001 | *R* = 0.5957  *p* < 0.0001 | *R* = 0.5530  *p* < 0.0001 | *R* = 0.5969  *p* < 0.0001 | *R* = 0.5352  *p* < 0.0001 | *R* = 0.5348  *p* < 0.0001 |

ADOS, autism diagnostic observation schedule; GCN, graph convolutional network; AAL, automated anatomical labelling.

**Table S5. Summary of previous studies that predicted ASD severity using brain MRI parameters**

| **Publication** | **Datasets** | **Number of Cases** | **Case Ages** | **Clinical index** | **Neuroimaging Modalities** | **Processing Toolbox** | **Machine-learning Methods** | **Identified regions** | **Performance** |
| --- | --- | --- | --- | --- | --- | --- | --- | --- | --- |
| Pua et al., 2021 (Cerebral Cortex) | ABIDE-II | 100 ASD, 100 TD | Avg. 11.43y | SRS | T1-weighted MRI, rs-fMRI | CONN, SPM12 | Non-negative matrix factorization | Salience network and occipital temporal face perception network | Subject-level distance-based approach: t = 2.206 *(p = 0.030)* |
| Souza et al., 2020 (NeuroImage) | ABIDE-I | 1^st^: 58 ASD, 2^nd^: 63 ASD | Avg. 10.06y | ADOS, SRS, Praxis | rs-fMRI | AFNI, SPM | JNO Framework | 8 subnetworks  (including DMN, SMN, visual processing areas, central executive control network) | ADOS-MAE: 2.53, SRS-MAE: 13.27, Praxis-MAE: 10.18 |
| Liu and Huang, 2020 (Scientific Reports) | ABIDE-I | 174 ASD | Avg. 16.74 | ADOS, GOTHAM_severity | rs-fMRI | AFNI | SVR | Top 27 regions  (SMN and DMN-related region, temporal and frontal lobe, language system related ROI like Heschl’s gyri, STG, MTG, left Rolandic operculum) | R = 0.50 *(p < 0.0001),* MAE = 1.41 |
| Pua et al., 2019 (Scientific Reports) | ABIDE-II | 100 ASD, 100 TD | Avg. 11.43y | SRS | T1-weighted MRI | Freesurfer | Elastic Net | Salience network, DMN  (widespread, 19 cortical thickness and 10 surface area features) | R^2^ = 0.153 *(p = 0.01)* |
| Lake et al., 2019 (Biological Psychiatry) | ABIDE-I, ABIDE-II, ADHD-200 | 290 ASD, 342 TD | 7-64 y | ADOS, SRS, | fMRI | AFNI, SPM8 | Connectome-based predictive modeling | Default mode, limbic, visuospatial, motor, subcortical, and cerebellum | R (SRS): 0.32 *(p < 0.00002)*  R (ADOS): 0.43 (*p < 0.0002)* |
| Moradi et al., 2017 (NeuroImage) | ABIDE | 156 ASD | 7-64 y | ADOS proxy calibrated severity score | T1-weighted MRI (cortical thickness) | CIVET | SVR, Elastic Net LR | Top 24 regions  Language related (pars triangularis, rolandic operculum, STG, angular gyrus), mirror neuron system (intraparietal sulci), social orienting/joint attention (ACC, DMFC) , repetitive behaviors (basal ganglia, SMA) | R (ADOS): 0.51 *(p < 0.0001)*, MAE: 1.34 |
| Yahata et al., 2016  (Nature communications) | Clinical Acquisition | 74 ASD, 107 TD | Avg. 30.68y | ADOS, ADI-R | rs-fMRI | SPM8 | *L*_1_-SCCA and SLR | 16 abnormal functional connections (including cingulo-opercular network) | R (ADOS): 0.44 *(p < 0.001)* |
| Sato et al., 2013 (J of Psychiatric Research) | Clinical Acquisition | 82 ASD, 84 TD | 18-42y | ADOS | T1-weighted MRI | Freesurfer | SVR | Top 10 regions (pars triangularis, postcentral, caudal middle frontal, temporal pole, frontal pole, entorhinal, STS) | R (ADOS): 0.362 *(p < 0.001)* |
| Uddin et al., 2013 (JAMA Psychiatry) | Clinical Acquisition | 20 ASD, 20 TD | 7–12y | ADOS, ADI-R | rs-fMRI | FSL, SPM8 | GLMnet | Salience network (including anterior insula and ACC) | R^2^ (ADIR): 0.36 (*p =* 0.007) |

ASD, autism spectrum disorder; MRI, magnetic resonance imaging; ABIDE, autism brain imaging data exchange; TD, typical development; SRS, social responsiveness scale; fMRI, functional magnetic resonance imaging; rs-fMRI, resting state functional magnetic resonance imaging; ADOS, autism diagnostic observation schedule; JNO, joint network optimization; DMN, default mode network; SMN, sensorimotor network; MAE, mean absolute error; SVR, support vector regression; ROI, region of interest; STG, superior temporal gyrus; MTG, middle temporal gyrus; ADHD, attention deficit hyperactivity disorder; LR, linear regression; ACC, anterior cingulate cortex; DMFC, dorsomedial frontal cortex; SMA, supplementary motor area; *L*_1_-SCCA, *L*_1_-norm regularized sparse canonical correlation analysis; SLR, sparse logistic regression; ADI-R, autism diagnostic interview-revised;.

**Figure legends**

**Figure S1. The flow chart for data preparation.**

**Figure S2. Prediction performances for the proposed model with the FIND atlas**. Comparison of the ASD prediction results for benchmarking the BrainNetCNN model (Kawahara et al., 2017; column (a)) and different configurations of proposed model (column (b)-(d)) under two functional connectivity settings based on the Pearson correlation coefficient (diamond markers, and dashed line of best fit) and the Tikhonov covariance (circle markers, and solid line of best fit) with the FIND atlas. The colors of the markers and lines indicate the increase (red) or decrease (gray) in the MAE of the prediction as compared with the benchmarking model.

**Figure S3. Prediction performances for the proposed model with the MMP atlas**. Comparison of the ASD prediction results for the benchmarking BrainNetCNN model (Kawahara et al., 2017; column (a)) and different configurations of proposed model (column (b)-(d)) under two functional connectivity settings based on the Pearson correlation coefficient (diamond markers, and dashed line of best fit) and the Tikhonov covariance (circle markers, and solid line of best fit) with the MMP atlas. The colors of the markers and lines indicate the increase (red) or decrease (gray) in the MAE of the prediction as compared with the benchmarking model.

**Figure S4. Prediction performances for the proposed model with the SHEN atlas**. Comparison of the ASD prediction results for benchmarking BrainNetCNN model (Kawahara et al., 2017; column (a)) and different configurations of proposed model (column (b)-(d)) under two functional connectivity settings based on the Pearson correlation coefficient (diamond markers, and dashed line of best fit) and the Tikhonov covariance (circle markers, and solid line of best fit) with the SHEN atlas. The colors of the markers and lines indicate the increase (red) or decrease (gray) in the MAE of the prediction as compared with the benchmarking model.

**Movie S1 (separate file). A graphic video of the identified contributive functional connections for prediction of ASD severity.** The video started from the right temporal lobe and moves to the frontal, left temporal, and occipital lobe, and finished at both hemispheres. The first and last phases showed the most predictive connection labels trained by our proposed model; ADOS severity (red), ADI RRB (pink), ADI verbal (yellow), and ADI social (Blue). For each predictive connection that accounts for the severity score, the nodes that have the intensity of top 10% magnitude (big nodes) and 20% magnitude (small nodes) were shown. The thickness of the edges reflected the contribution values of predicting ASD scores.
