# Supplementary figures and images for: Sparse Hierarchical Representation Learning on Functional Brain Networks for Prediction of Autism Severity Levels
Source: Front Neurosci. 2022 Jul 7;16:935431. doi: 10.3389/fnins.2022.935431 (PMC9301472; doi:10.3389/fnins.2022.935431)

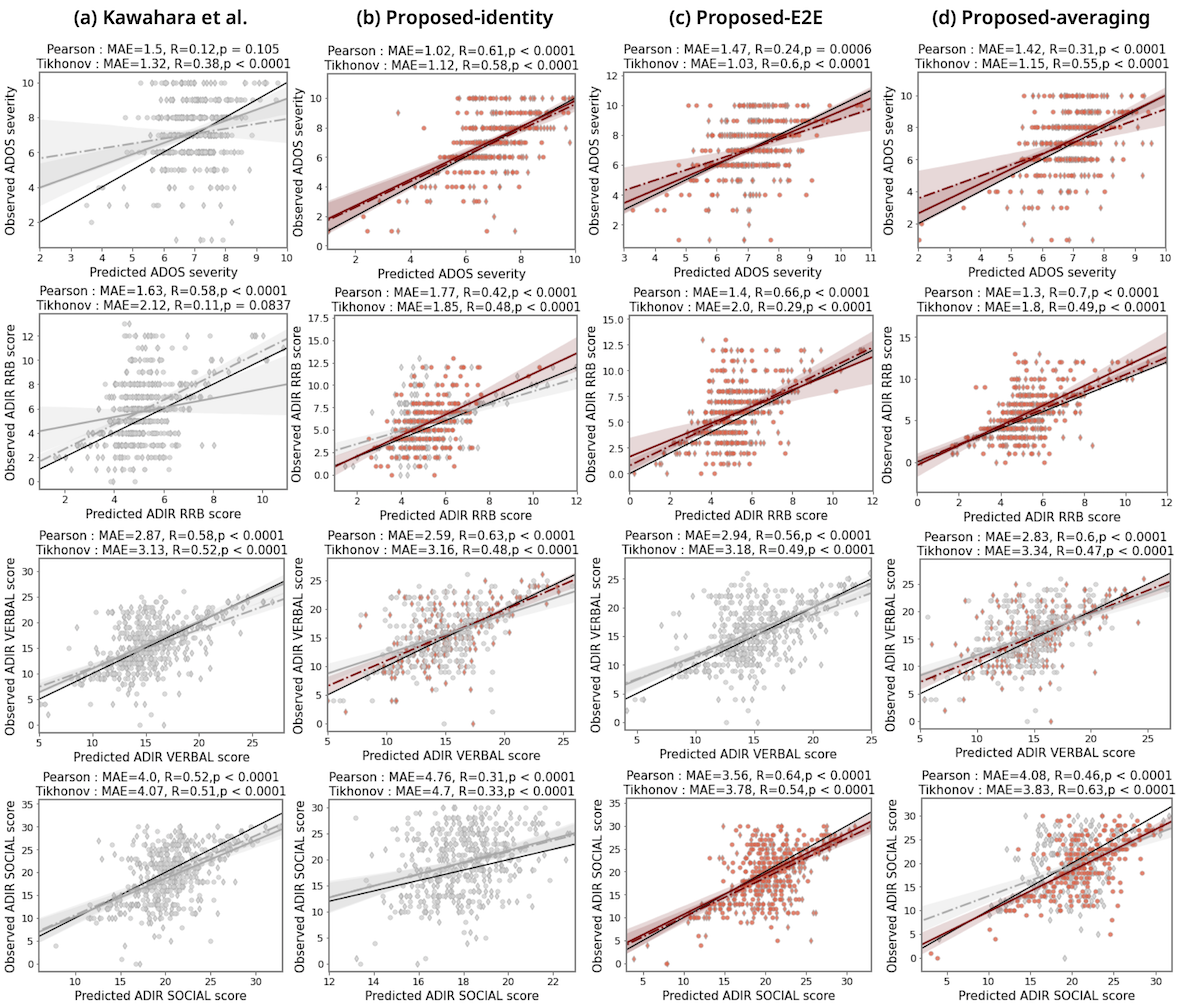

Supplement: Supplementary file 3 [file Image_1.TIFF]

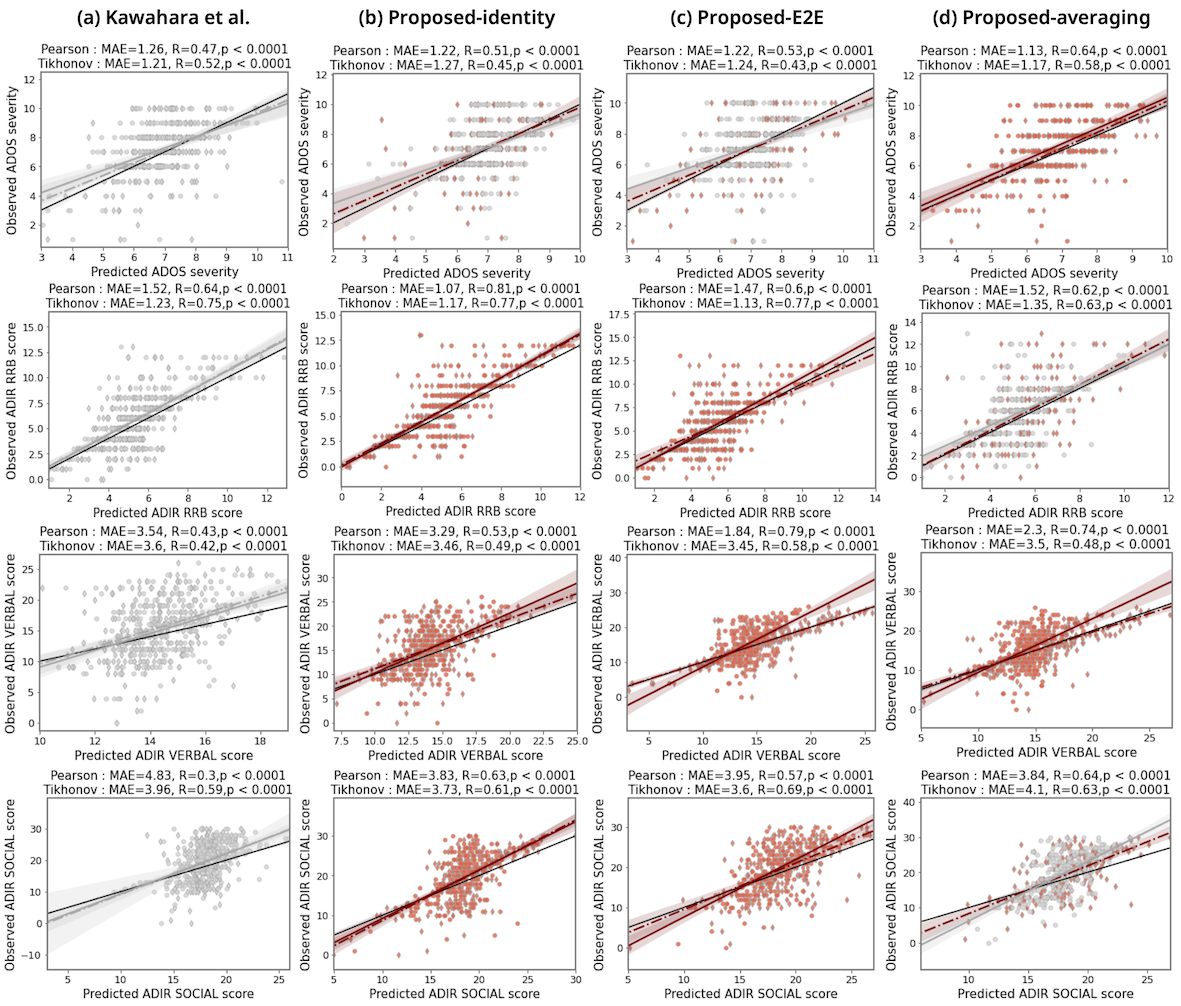

Supplement: Supplementary file 4 [file Image_2.TIFF]

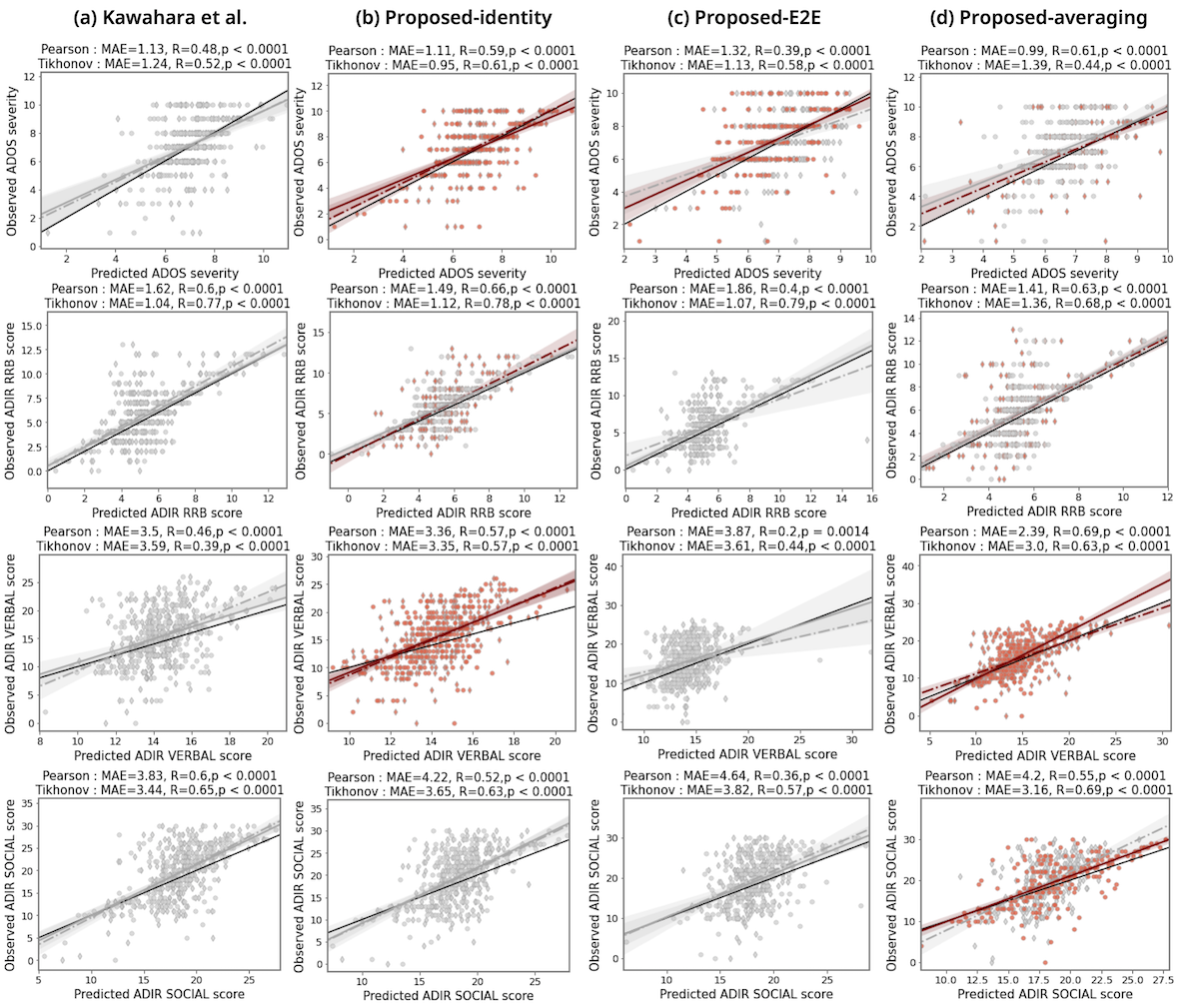

Supplement: Supplementary file 5 [file Image_3.TIFF]
